# Temporal epidemiology and multi-source forecasting of hemorrhagic fever with renal syndrome and leptospirosis in mainland China: An interpretable machine learning study

**DOI:** 10.1016/j.pmedr.2026.103643

**Published:** 2026-09-17

**Authors:** Meiqian Gong, Yinzhu Zhou, Shuilian Chen, Chi Zhang

**Affiliations:** a Changsha Municipal Center for Disease Control and Prevention, Changsha 410004, China

**Keywords:** Hemorrhagic fever with renal syndrome, Leptospirosis, Time-series forecasting, Multi-source data, Interpretable machine learning

## Abstract

**Objective:**

Hemorrhagic fever with renal syndrome (HFRS) and leptospirosis are environmentally associated zoonoses in China, yet their long-term temporal dynamics and predictive determinants have not been systematically compared.

**Methods:**

We analyzed monthly surveillance data on reported cases of HFRS and leptospirosis in mainland China from January 2010 to December 2025 using a multi-source data-driven framework. Five statistical, machine-learning, and deep-learning models were evaluated using historical, socioeconomic, meteorological, ecological, and behavioral predictors.

**Results:**

During the study period, 152,183 HFRS cases and 6288 leptospirosis cases were reported. HFRS declined significantly after 2019, whereas leptospirosis declined until 2018 and subsequently rebounded. Both diseases exhibited distinct seasonality, with HFRS peaking in late autumn and winter and leptospirosis in late summer and autumn. Extreme gradient boosting (XGBoost) with the combined predictor set achieved the best out-of-sample performance for both diseases. Shapley additive explanations identified 12-month-lagged incidence as the dominant predictor for both diseases, whereas precipitation and relative humidity contributed more strongly to leptospirosis than to HFRS.

**Conclusions:**

HFRS and leptospirosis showed distinct epidemiological patterns and predictor contributions. Integrating multi-source predictors improved forecasting performance, while interpretable machine learning provided disease-specific insights to support early warning and targeted prevention of environmentally associated zoonoses.

## Highlights

• Systematic comparison of hemorrhagic fever with renal syndrome and leptospirosis.
• Multi-source predictors improved forecasting for both zoonotic diseases.
• Extreme gradient boosting achieved the best performance among five models.
• Shapley additive explanations revealed shared and disease-specific contributions.
• The framework supports interpretable early warning for zoonotic diseases.

## 1 Introduction

Hemorrhagic fever with renal syndrome (HFRS) and leptospirosis are important environmentally associated zoonotic diseases. Hantavirus infections cause an estimated 10,000 to more than 100,000 cases annually worldwide, particularly in Asia and Europe (Note, n.d.), whereas leptospirosis causes approximately 1.03 million cases and 58,900 deaths each year (Costa et al., 2015). In China, HFRS has remained an important rodent-borne zoonosis over the past two decades, while 4559 leptospirosis cases and 49 deaths were reported during 2010–2022 (Zhai et al., 2026; Shi et al., 2024). Although their incidence has declined in many areas, persistent natural foci and changing environmental and exposure conditions continue to challenge prevention and control (Zhai et al., 2026; Shi et al., 2024; Wang et al., 2025).

HFRS and leptospirosis share ecological features but differ in transmission. Both are influenced by reservoir hosts, climate, ecological settings, and human activity (Note, n.d.; Taylor et al., 2025; Bradley and Lockaby, 2023; Antoniolli et al., 2025). HFRS is mainly transmitted through exposure to infected rodents or their excreta (Note, n.d.; Taylor et al., 2025), whereas leptospirosis is commonly linked to water, soil, or animal urine contaminated with pathogenic *Leptospira* spp. (Bradley and Lockaby, 2023; Antoniolli et al., 2025). These shared environmental dependencies and distinct pathways may produce common and disease-specific temporal patterns and predictors, making comparative analysis useful for early warning and targeted interventions (Zhang et al., 2024; Basheer et al., 2025).

Previous studies have described the epidemiological characteristics and seasonality of HFRS or leptospirosis, and traditional time-series models have been used for short-term forecasting (Gao et al., 2023; Lv et al., 2021; Govan et al., 2025). However, most studies focused on a single disease or relied mainly on historical incidence, limiting their ability to capture nonlinear effects from multi-source external predictors (Gao et al., 2023; Govan et al., 2025; Kraemer et al., 2025). In addition, machine learning models have increasingly been applied to infectious disease prediction, but their limited interpretability remains a major barrier to public health application (Kraemer et al., 2025; Joyce et al., 2023). A systematic framework integrating multi-source predictors with interpretable machine learning is therefore needed to improve both forecasting performance and understanding of disease determinants.

In this study, we analyzed monthly reported HFRS and leptospirosis cases in mainland China from 2010 to 2025. We first characterized their long-term trends, seasonality, temporal dependence, and periodicity. We then developed and compared statistical, machine-learning, and deep-learning forecasting models using historical, socioeconomic, meteorological, ecological, and behavioral predictors. Finally, we applied Shapley additive explanations (SHAP) to quantify the relative contributions of key predictors and predictor domains. Overall, this study provides a systematic assessment of the temporal dynamics, forecastability, and contributions of multi-source predictors for HFRS and leptospirosis in China.

## 2 Methods

### 2.1 Study design and population

This study analyzed monthly surveillance data on reported cases of HFRS and leptospirosis in mainland China from January 2010 to December 2025. The data were obtained from the National Bureau of Disease Control and Prevention of China (https://www.ndcpa.gov.cn). The study area comprised 31 provincial-level administrative divisions, excluding the Hong Kong and Macao Special Administrative Regions and Taiwan, China. Cases were reported through China's national notifiable infectious disease surveillance system, which operates under standardized reporting and quality-control procedures.

### 2.2 Measures

To systematically characterize disease determinants, predictors from five domains were collected: (1) historical indicators, including lagged case counts, rolling averages, and seasonal terms; (2) socioeconomic variables from the National Bureau of Statistics of China (https://data.stats.gov.cn/); (3) meteorological variables, including temperature, relative humidity, and precipitation, obtained from the Copernicus Climate Data Store (Copernicus Climate Change Service, Climate Data Store, 2023); (4) ecological variables derived from Google Earth Engine and national statistical datasets (Gorelick et al., 2017); and (5) behavioral variables, including passenger transportation, holiday indicators, and Baidu Search Index data. Gridded environmental variables were spatially aggregated to the national level using population-weighted averaging. Candidate predictors were screened using training-period data based on pairwise correlations (|*r*| ≥ 0.70), variance inflation factors (VIFs; retained values <10), and epidemiological relevance. Correlation and VIF results before and after screening are provided in Supplementary Table S1.

To evaluate the incremental contribution of each predictor domain, six predictor sets were constructed. M0 was the baseline historical predictor set and included lagged case counts, a 12-month rolling mean, and sine and cosine transformations of calendar month; M1, M2, M3, and M4 separately extended M0 by adding socioeconomic, meteorological, ecological, and behavioral predictors, respectively. M5 integrated selected predictors from all five domains. Because feature selection was conducted separately for HFRS and leptospirosis, the final predictor composition differed slightly between the two diseases. Detailed predictor definitions, lag structures, and the number of predictors included in each set are provided in Supplementary Table S2.

### 2.3 Statistical analysis

#### 2.3.1 Temporal epidemiological analysis

Temporal characteristics of HFRS and leptospirosis were evaluated using complementary descriptive and time-series analyses. Annual trends were quantified using Joinpoint regression (Liu et al., 2026) to estimate annual percentage changes (APCs). Seasonal-trend decomposition using locally estimated scatterplot smoothing (STL) was performed to separate long-term trends and seasonal components. Temporal dependence within each disease was assessed using autocorrelation function analysis, while cross-correlation function analysis was used to evaluate temporal associations between the two diseases. Wavelet power spectrum and wavelet coherence analyses were applied to the monthly case-count series for 2010–2025 after natural-log transformation and separate z-standardization of each disease series to a mean of 0 and a standard deviation of 1 (Torrence and Compo, 1998; Grinsted et al., 2004).

#### 2.3.2 Forecasting models

Five forecasting approaches were evaluated, including seasonal autoregressive integrated moving average (SARIMA), random forest, extreme gradient boosting (XGBoost), long short-term memory (LSTM), and temporal convolutional network (TCN) (Nixon et al., 2022; Ye et al., 2025; Chen and Guestrin, 2016; Zhou et al., 2023; Li et al., 2023). Each disease series comprised 192 monthly observations: 162 from January 2010 to June 2023 were used for model training, and 30 from July 2023 to December 2025 were reserved for independent testing.

SARIMA was fitted using historical incidence only (M0). Random forest and XGBoost were trained using all predictor sets (M0–M5), with hyperparameters optimized by iterative grid search using five-fold expanding-window time-series cross-validation with 12-month validation blocks. LSTM and TCN used a fixed 12-month look-back window, with hyperparameters selected by grid search using the final 20% of the training sequences as a chronological validation set and early stopping. All tuning was restricted to the training period. The optimized configurations corresponding to the best-performing predictor set for each model and disease are provided in Supplementary Table S3.

#### 2.3.3 Model evaluation

Forecasting performance was evaluated in both the training and independent test periods using root mean squared error (RMSE) as the primary metric. Mean absolute error (MAE) and coefficient of determination (*R*^2^) were used as secondary metrics. Training-set metrics were calculated after excluding the initial 12-month burn-in period, whereas test-set metrics were based on 30 rolling one-month-ahead forecasts.

#### 2.3.4 Shapley additive explanation analysis

The best-performing model was interpreted using SHAP, a game theory-based approach that quantifies the contribution of individual predictors to model predictions (Lundberg and Lee, 2017). For a given observation, the SHAP explanation model can be expressed as:

$$g\left(z^{\prime }\right)=\varphi_{0}+\sum_{j=1}^{M}\varphi_{j}z_{j}^{\prime }$$

where $g\left(z^{\prime }\right)$ denotes the explanation model, $\varphi_{0}\;$is the baseline model output, *M* is the number of predictors, $\varphi_{j}\;$represents the SHAP value of predictor *j*, and $z^{\prime }\in {\left\{01\right\}}^{M}\;$is the coalition vector of simplified features, where 1 means the feature is present, and 0 means it is absent in the coalition. A positive SHAP value indicates that the predictor increases the predicted number of cases relative to the baseline, whereas a negative value indicates the opposite (Lundberg and Lee, 2017).

Mean absolute SHAP values were used to rank predictor importance and were further aggregated into five predictor domains (historical, socioeconomic, meteorological, ecological, and behavioral) to evaluate their relative contributions. Predictor importance was also compared across different epidemic phases defined by Joinpoint regression.

#### 2.3.5 Software

Joinpoint regression was performed using the Joinpoint Regression Program (National Cancer Institute, Bethesda, Maryland, United States). All other analyses were conducted in Python 3.11 using pandas and NumPy for data management, statsmodels for time-series analysis, scikit-learn and XGBoost for machine learning, PyTorch for deep learning, PyCWT for wavelet analysis, SHAP for model interpretation, and Matplotlib for visualization.

### 2.4 Ethics statement

This study was a secondary analysis of publicly available, aggregated routine surveillance data and involved no individual-level or identifiable information. The study complied with applicable data-use and privacy requirements. The Ethics Committee of the Changsha Municipal Center for Disease Control and Prevention confirmed that the study was exempt from ethical review and waived the requirement for individual informed consent.

## 3 Results

### 3.1 Temporal epidemiology

#### 3.1.1 National trends

From January 2010 to December 2025, 152,183 HFRS cases and 6288 leptospirosis cases were reported in mainland China. The monthly case burden of HFRS was consistently higher than that of leptospirosis throughout most of the study period (Fig. 1A). However, the two diseases showed distinct long-term temporal trends. HFRS remained at a relatively high level during the early years of the study before declining markedly after 2019, whereas leptospirosis decreased until 2018 and subsequently rebounded.

**Fig. 1:**
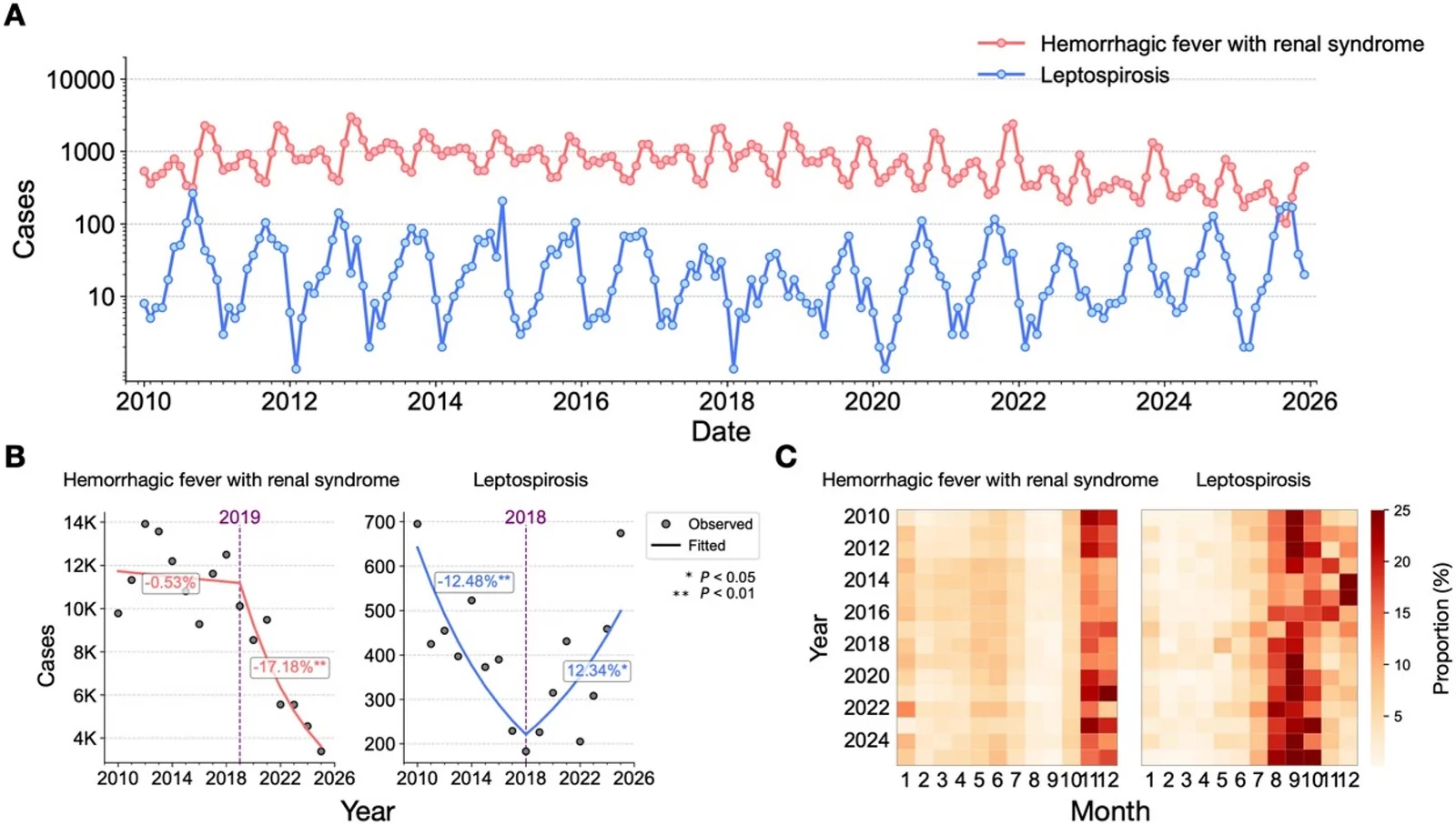
**Temporal trends and seasonal distribution of monthly reported cases of hemorrhagic fever with renal syndrome and leptospirosis in mainland China, January 2010–December 2025.** (A) Monthly reported cases of hemorrhagic fever with renal syndrome and leptospirosis. The y-axis is presented on a logarithmic scale. (B) Joinpoint regression of annual case counts. Points indicate observed values, solid lines indicate fitted trends, and dashed vertical lines indicate identified joinpoints. Annual percentage changes are shown for each trend segment. Asterisks indicate statistical significance: *P* < 0.05 (*) and *P* < 0.01 (**). (C) Heatmaps showing monthly cases as percentages of the corresponding annual case counts. Darker colors indicate higher seasonal concentration.

Joinpoint regression confirmed these divergent trends (Fig. 1B). For HFRS, 2019 was identified as the major turning point. Annual case numbers remained relatively stable before 2019 (APC = −0.53%, *P* = 0.86) but declined significantly thereafter (APC = −17.18%, *P* < 0.01). In contrast, leptospirosis declined significantly during 2010–2018 (APC = −12.48%, *P* < 0.01) and increased significantly during 2018–2025 (APC = 12.34%, *P* < 0.05).

Distinct seasonal patterns were also observed (Fig. 1C). HFRS cases were concentrated mainly in late autumn and winter, particularly in November and December, whereas leptospirosis showed a consistent peak during late summer and autumn, especially between August and October.

#### 3.1.2 Seasonality and temporal dependence

HFRS and leptospirosis both exhibited pronounced seasonality but differed in temporal structure (Fig. 2). STL decomposition showed strong seasonal components for both HFRS and leptospirosis, although HFRS displayed a stronger long-term trend component (Supplementary Fig. S1).

**Fig. 2:**
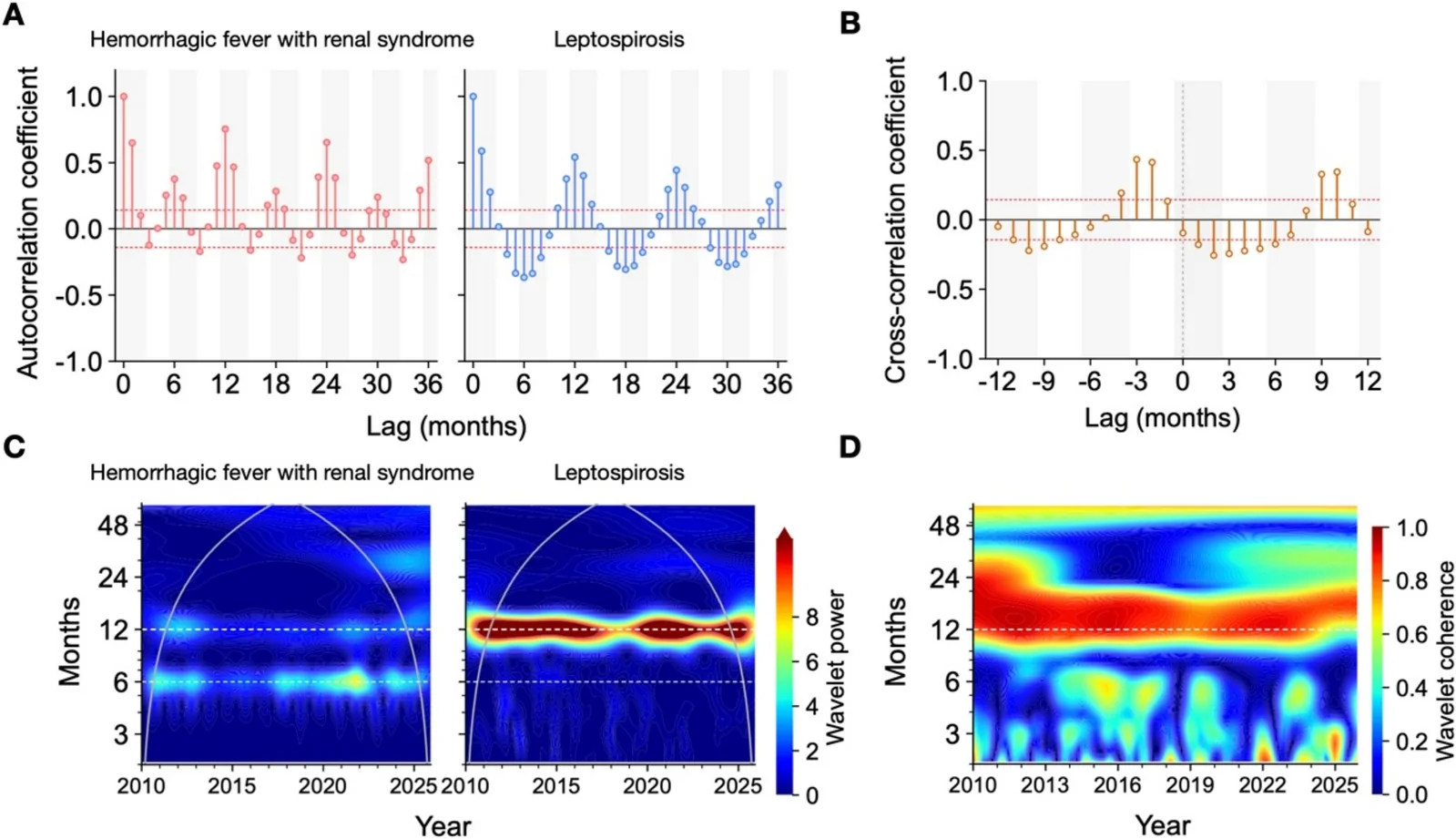
**Temporal dependence, periodicity, and shared time-frequency structures of monthly reported cases of hemorrhagic fever with renal syndrome and leptospirosis in mainland China, January 2010–December 2025.** (A) Autocorrelation functions of monthly reported hemorrhagic fever with renal syndrome and leptospirosis cases up to 36 monthly lags. Dashed horizontal lines indicate the approximate 95% confidence limits. (B) Cross-correlation function between the two diseases at lags from −12 to +12 months. Dashed horizontal lines indicate the approximate 95% confidence limits, and the vertical dotted line indicates zero lag. Positive lags indicate that hemorrhagic fever with renal syndrome precedes leptospirosis, whereas negative lags indicate the reverse. (C) Wavelet power spectra of the log-transformed and standardized monthly case-count series. Warmer colors indicate stronger wavelet power, dashed horizontal lines indicate 6- and 12-month reference periods, and curved white lines indicate the cone of influence. (D) Wavelet coherence between the two diseases, showing their shared time-frequency structure. Warmer colors indicate stronger coherence, and the dashed horizontal line indicates the 12-month reference period.

Autocorrelation analysis demonstrated significant short-term and annual temporal dependence for both diseases, supporting the inclusion of lagged incidence and seasonal variables in forecasting models (Fig. 2A). Wavelet power spectra further revealed different periodic characteristics. HFRS exhibited a more complex and time-varying periodic pattern, with dominant signals around 5.84 months and intermittent annual cycles, whereas leptospirosis maintained a stable annual cycle centered at approximately 11.69 months (Fig. 2C).

Although the two diseases showed weak synchrony at zero lag, cross-correlation analysis identified the strongest positive association at a lag of −3 months (Fig. 2B). Wavelet coherence analysis further demonstrated strong coherence within the annual frequency band (10–14 months), indicating that the two diseases shared similar annual-scale periodicity despite differences in seasonal timing (Fig. 2D).

### 3.2 Forecasting performance

Forecasting performance differed substantially by model type and predictor set (Table 1 and Supplementary Table S4). For HFRS, XGBoost-M5 achieved the lowest test-set RMSE (125.88), followed by random forest-M5 (151.54), TCN-M2 (166.65), LSTM-M0 (180.48), and SARIMA-M0 (187.50). The selected SARIMA benchmark was SARIMA(1,0,1)(0,1,1)_12_. Compared with SARIMA-M0 and XGBoost-M0, XGBoost-M5 reduced the test RMSE by 32.86% and 31.00%, respectively, demonstrating the benefits of nonlinear multi-source modeling and the added value of multi-source predictors.

**Table 1:** Training- and test-set root mean square error of forecasting models across predictor sets for monthly reported cases of hemorrhagic fever with renal syndrome and leptospirosis in mainland China, January 2010–December 2025.

Disease	Model	Dataset	M0 (historical predictors)	M1 (historical and socioeconomic predictors)	M2 (historical and meteorological predictors)	M3 (historical and ecological predictors)	M4 (historical and behavioral predictors)	M5 (all predictor domains)
Hemorrhagic fever with renal syndrome	Seasonal autoregressive integrated moving average	Training	183.74	–	–	–	–	–
		Test	187.50	–	–	–	–	–
	Random forest	Training	177.56	175.19	150.57	182.74	159.19	146.43
		Test	186.91	176.04	162.22	185.83	176.16	151.54
	Extreme gradient boosting	Training	179.71	139.85	129.19	151.60	164.03	116.02
		Test	182.44	170.62	134.09	178.33	171.55	125.88
	Long short-term memory	Training	142.89	170.37	144.83	149.49	191.15	193.46
		Test	180.48	440.87	247.51	241.26	353.65	456.28
	Temporal convolutional network	Training	111.10	154.68	104.94	103.16	149.69	170.30
		Test	199.44	390.61	166.65	180.83	171.59	295.00

Leptospirosis	Seasonal autoregressive integrated moving average	Training	26.31	–	–	–	–	–
		Test	28.09	–	–	–	–	–
	Random forest	Training	15.95	15.53	14.02	15.69	14.13	13.60
		Test	32.83	30.14	26.52	32.00	28.10	22.06
	Extreme gradient boosting	Training	14.31	12.53	10.68	14.26	12.76	8.73
		Test	29.05	26.17	21.40	26.84	22.45	14.91
	Long short-term memory	Training	20.77	22.46	19.21	21.93	19.20	17.25
		Test	37.69	48.36	32.03	46.68	46.14	54.61
	Temporal convolutional network	Training	20.28	21.92	17.54	22.80	18.33	21.30
		Test	35.20	42.01	31.90	45.37	42.45	37.85

Note: Lower root mean square error values indicate better predictive performance. M0 included historical predictors only; M1 included historical and socioeconomic predictors; M2 included historical and meteorological predictors; M3 included historical and ecological predictors; M4 included historical and behavioral predictors; and M5 included all five predictor domains. Seasonal autoregressive integrated moving average was evaluated only under M0; dashes indicate not applicable.

For leptospirosis, XGBoost-M5 also showed the best out-of-sample performance, with the lowest RMSE (14.91), followed by random forest-M5 (22.06), SARIMA-M0 (28.09), TCN-M2 (31.90), and LSTM-M2 (32.03). The selected SARIMA benchmark was SARIMA(0,1,1)(0,1,1)_12_. Compared with SARIMA-M0 and XGBoost-M0, XGBoost-M5 reduced the test RMSE by 46.92% and 48.67%, respectively, showing the same benefits of nonlinear modeling and multi-source predictors.

The fitted and forecast trajectories further supported these findings (Fig. 3). SARIMA captured the broad seasonal pattern but showed limited flexibility in tracking changing peak amplitudes. Random forest more closely tracked the observed cases but smoothed some high-incidence peaks. XGBoost provided the closest agreement with observed cases during the independent test period for both HFRS and leptospirosis. LSTM and TCN showed less stable out-of-sample performance, especially for HFRS, indicating that deep learning models did not outperform the tree-based models under the current national monthly dataset. Overall, XGBoost-M5 provided the most accurate and stable forecasts for both diseases and was therefore selected for subsequent model interpretation.

**Fig. 3:**
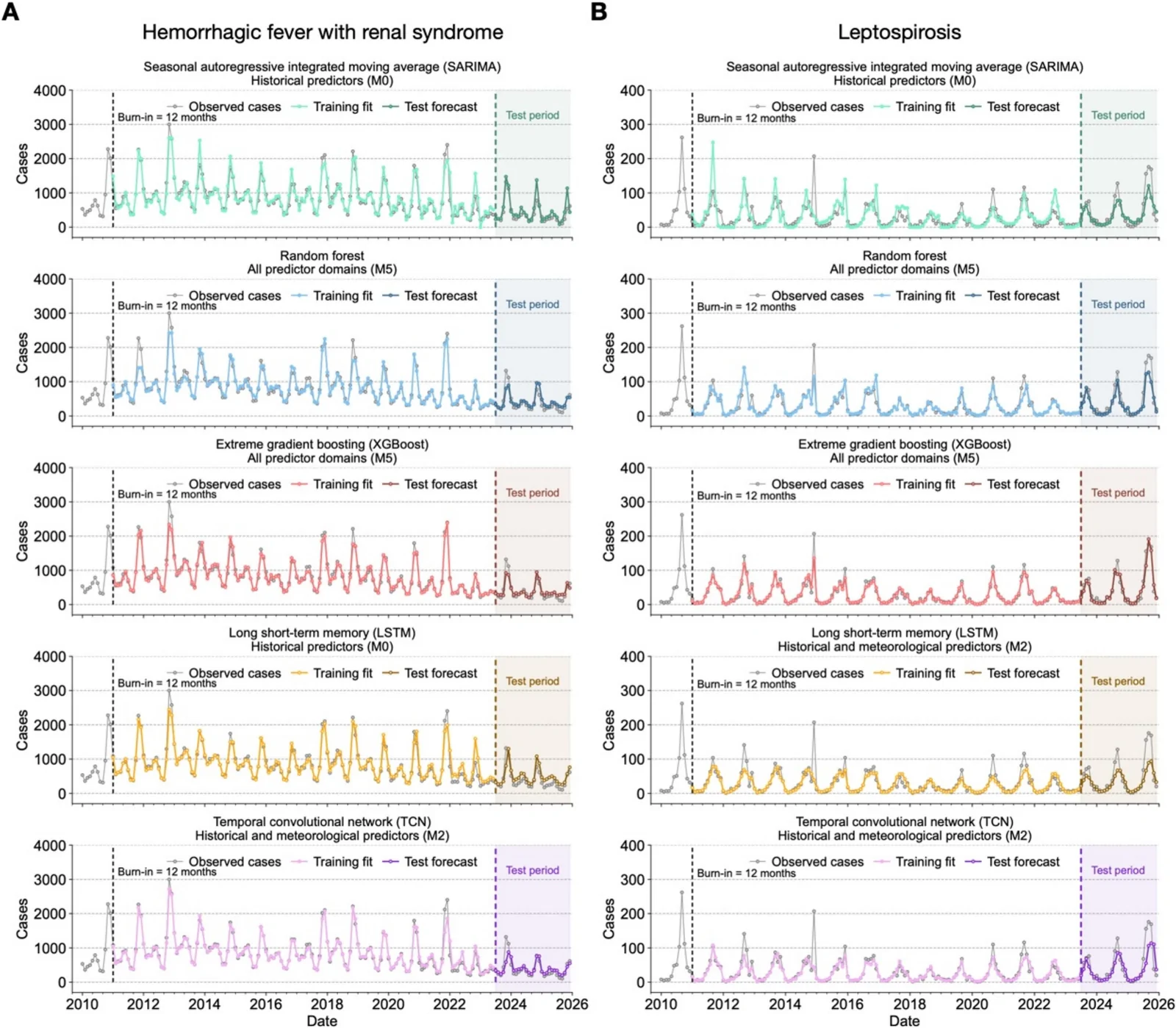
**Observed, fitted, and forecast monthly reported cases of hemorrhagic fever with renal syndrome and leptospirosis across forecasting models in mainland China, January 2010–December 2025.** (A) Observed, fitted, and forecast monthly cases of hemorrhagic fever with renal syndrome using the predictor set with the lowest test-set root mean square error for each model. (B) Corresponding results for leptospirosis. Grey lines represent observed cases, light-colored lines represent fitted values during the training period, and dark-colored lines represent rolling one-month-ahead forecasts during the independent test period. The first vertical dashed line marks the end of the 12-month burn-in period, after which training-period fitted values are displayed. The second vertical dashed line and the shaded region mark the independent test period from July 2023 through December 2025. M0 included historical predictors only; M2 included historical and meteorological predictors; and M5 included all five predictor domains. SARIMA, seasonal autoregressive integrated moving average; XGBoost, extreme gradient boosting; LSTM, long short-term memory; TCN, temporal convolutional network.

### 3.3 Predictor contributions

Given the superior forecasting performance of XGBoost-M5, SHAP analysis was used to quantify the contribution of multi-source predictors to model outputs (Fig. 4). For HFRS, historical predictors contributed most to model predictions, with HFRS incidence at lags of 12 and 1 months accounting for 35.27% and 15.25% of the total absolute SHAP contribution, respectively (Fig. 4A). Other important predictors included relative humidity at lag 3, school holidays, the Baidu Index for rodent infestation at lag 1, HFRS incidence at lag 6, and the 12-month rolling mean of HFRS incidence. At the domain level, historical factors accounted for the largest contribution (60.76%), followed by behavioral (18.11%), meteorological (8.94%), socioeconomic (8.85%), and ecological factors (3.34%). These results indicate that the HFRS forecasts were primarily driven by historical incidence patterns, with additional contributions from behavioral, meteorological, socioeconomic, and ecological predictors.

**Fig. 4:**
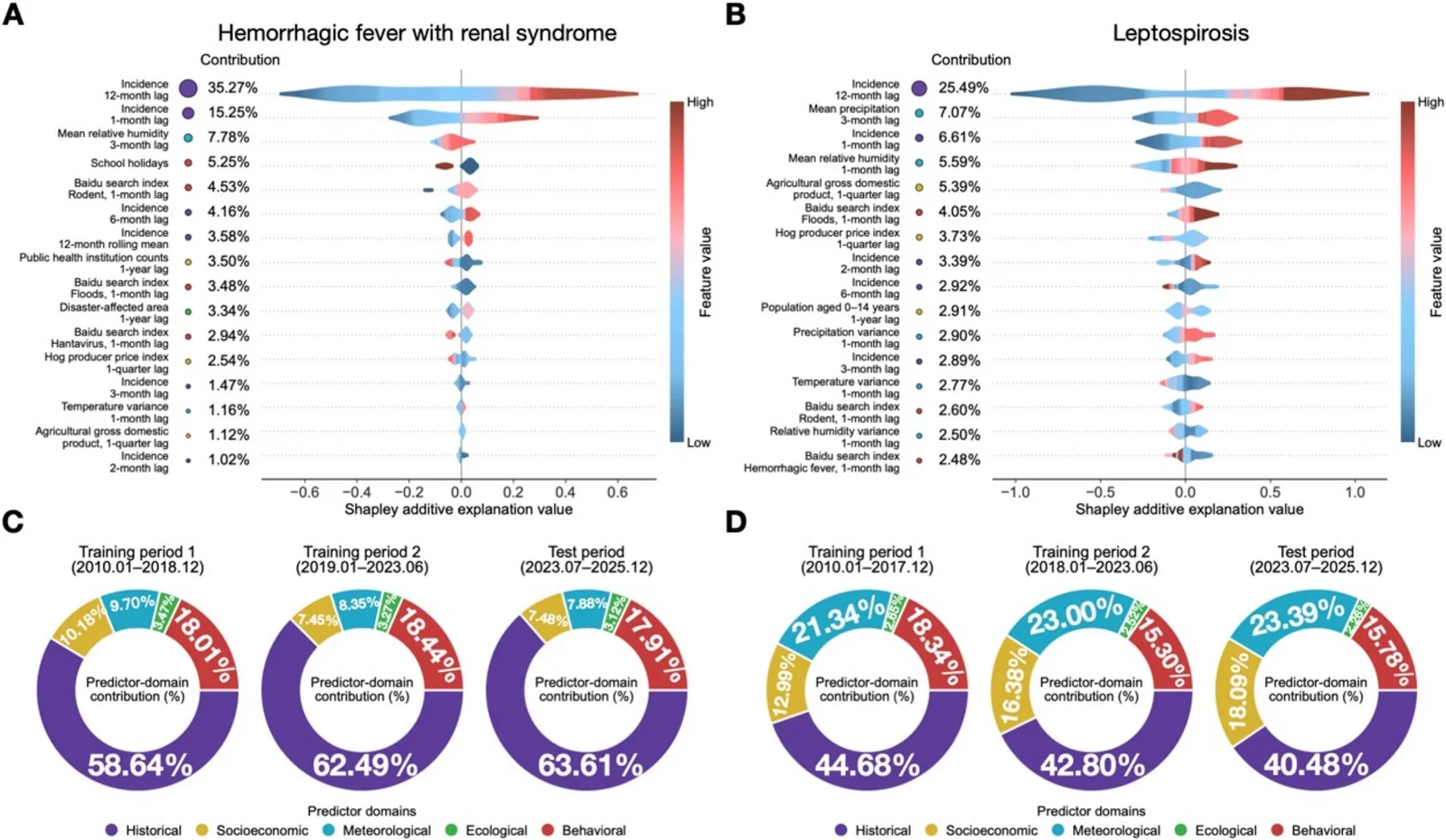
**Contributions of multi-source predictors to forecasts of monthly reported cases of hemorrhagic fever with renal syndrome and leptospirosis in mainland China, January 2010–December 2025.** (A) Summary plot based on Shapley additive explanation values for the extreme gradient boosting model using all predictor domains (M5) for hemorrhagic fever with renal syndrome. The 15 predictors with the largest mean absolute Shapley additive explanation values are shown. Percentages indicate each predictor's share of the total absolute contribution. Positive values indicate higher predicted monthly case counts relative to the model baseline, whereas negative values indicate lower predicted counts. Red and blue colors represent high and low predictor values, respectively. (B) Corresponding summary plot for leptospirosis. (C) Predictor-domain contributions for hemorrhagic fever with renal syndrome during training period 1 (January 2010–December 2018), training period 2 (January 2019–June 2023), and the test period (July 2023–December 2025). (D) Predictor-domain contributions for leptospirosis during training period 1 (January 2010–December 2017), training period 2 (January 2018–June 2023), and the test period (July 2023–December 2025). In panels C and D, percentages indicate each predictor domain's share of the total absolute contribution within the corresponding period. M5 combined selected historical, socioeconomic, meteorological, ecological, and behavioral predictors. (For interpretation of the references to colour in this figure legend, the reader is referred to the web version of this article.)

For leptospirosis, historical and meteorological predictors made the largest contributions (Fig. 4B). Leptospirosis incidence at lag 12 (25.49%), precipitation at lag 3 (7.07%), and leptospirosis incidence at lag 1 (6.61%) were the three most important predictors. At the domain level, historical predictors accounted for 43.36% of the total absolute SHAP contribution, followed by meteorological (22.24%), behavioral (16.90%), socioeconomic (14.97%), and ecological predictors (2.54%).

Phase-specific SHAP analysis showed that the relative contributions of the predictor domains changed over time (Fig. 4C and D). Historical predictors remained dominant across all epidemic phases for both diseases. For HFRS, the contribution of historical predictors increased from 58.64% in training period 1 to 62.49% in training period 2 and 63.61% in the test period. Meteorological contributions declined from 9.70% to 8.35% and 7.88%, respectively, whereas behavioral contributions remained relatively stable. For leptospirosis, the contribution of historical predictors decreased from 44.68% in training period 1 to 42.80% in training period 2 and 40.48% in the test period. In contrast, meteorological contributions increased from 21.34% to 23.00% and 23.39%, respectively, while socioeconomic contributions increased from 12.99% to 16.38% and 18.09%. Overall, the predictions for both diseases relied heavily on historical incidence, whereas meteorological predictors made a substantially greater contribution to leptospirosis predictions than to HFRS predictions.

## 4 Discussion

This study compared the temporal epidemiology, forecasting performance, and predictor contributions of HFRS and leptospirosis in mainland China over 16 years. Although both are environmentally associated zoonoses with pronounced temporal dependence and seasonality, they differed in long-term trends and predictor profiles. Among the five forecasting approaches evaluated, XGBoost-M5 achieved the best predictive performance for both diseases. SHAP analysis further indicated that historical predictors contributed most to predictions for both diseases, whereas meteorological predictors played a substantially greater role in leptospirosis prediction than in HFRS prediction.

The temporal analyses revealed important differences in the epidemiology of the two diseases. HFRS declined markedly after 2019, whereas leptospirosis showed a rebound following a prolonged decline. These contrasting trends are consistent with differences in their transmission ecology and disease control strategies. The decline in HFRS is likely attributable to sustained rodent control, vaccination in endemic areas, improvements in living conditions, and enhanced public health interventions (Su et al., 2024; Wang et al., 2024). In contrast, leptospirosis remains highly sensitive to environmental and climatic conditions and may re-emerge under favorable ecological settings despite a relatively low baseline incidence (Wang et al., 2025; Bradley and Lockaby, 2023; Thibeaux et al., 2024; Acosta-España et al., 2024). Seasonal analyses further supported these differences. HFRS peaked primarily during late autumn and winter, whereas leptospirosis showed a stable peak in late summer and autumn. The strong contribution of lagged incidence to both models is consistent with these recurrent seasonal patterns. Behavioral predictors provided additional information for HFRS forecasting, potentially reflecting calendar-related exposure patterns and public attention. The greater contribution of precipitation and humidity to leptospirosis forecasting is biologically plausible because environmental moisture promotes the survival of pathogenic *Leptospira* and increases opportunities for human exposure following flooding or agricultural activities (Bradley and Lockaby, 2023; Thibeaux et al., 2024; Acosta-España et al., 2024). Socioeconomic predictors provided more modest incremental information, whereas ecological predictors contributed least to both models, possibly because national aggregation obscured local heterogeneity and some indicators changed slowly over time; these limited predictive contributions should not be interpreted as evidence of epidemiological irrelevance. Together, these findings suggest that leptospirosis forecasting may benefit from climate-sensitive early warning indicators, whereas HFRS forecasting should emphasize historical incidence while incorporating relevant behavioral signals.

Our findings also highlight the value of integrating multi-source information into infectious disease forecasting. Both random forest and XGBoost achieved their best performance with M5 and outperformed the SARIMA benchmark, with XGBoost-M5 providing the lowest test-set RMSE for both diseases. These results are consistent with previous studies showing that environmental variables, human mobility, and internet search data provide complementary information beyond historical incidence alone (Phung et al., 2025; Vahedi et al., 2021; Nsoesie et al., 2021). In contrast, LSTM and TCN did not outperform tree-based models and showed relatively unstable performance, especially for HFRS. This observation is consistent with evidence suggesting that deep learning models may require larger datasets than conventional machine learning methods to learn robust temporal representations (Hollmann et al., 2025). The monthly national surveillance data used in this study contained fewer than 200 observations, and the addition of numerous predictors may have increased model complexity relative to the available sample size. Under these circumstances, tree-based ensemble models provide a practical balance among predictive performance, data requirements, and interpretability for infectious disease surveillance.

This study integrates descriptive epidemiology, multi-source forecasting, and explainable machine learning within a common framework for two environmentally associated zoonoses. SHAP quantified the contributions of individual predictors and predictor domains, supporting disease-specific interpretation of forecasts for public health surveillance.

Several limitations should be acknowledged. First, the analyses were conducted using nationally aggregated monthly surveillance data, which may mask substantial spatial heterogeneity in transmission dynamics, environmental suitability, and intervention effectiveness (Wang et al., 2024). Future studies should evaluate this framework at provincial or finer spatial scales. Second, surveillance data may be influenced by changes in reporting practices, diagnostic capacity, and healthcare-seeking behavior over time. Third, several potentially important determinants, including rodent density, rodent infection rates, vaccination coverage, occupational exposure, and local sanitation conditions, were unavailable at the national monthly level and therefore could not be incorporated directly into the models. Finally, although SHAP improves the interpretability of machine learning models, SHAP values do not establish causal relationships between predictors and disease occurrence, and the estimated contributions may still be affected by residual correlations among predictors (Lundberg and Lee, 2017; Ponce-Bobadilla et al., 2024).

## 5 Conclusions

HFRS and leptospirosis exhibit distinct temporal dynamics and predictor profiles despite their shared environmental associations. Integrating multi-source predictors with explainable machine learning substantially improved forecasting accuracy and clarified the relative contributions of disease-specific predictors. These findings support disease-specific surveillance and early warning strategies and may inform the development of data-driven public health forecasting tools.

## CRediT authorship contribution statement

**Meiqian Gong:** Writing – original draft, Visualization, Validation, Software, Resources, Methodology, Investigation, Formal analysis, Data curation. **Yinzhu Zhou:** Writing – review & editing, Validation, Data curation. **Shuilian Chen:** Writing – review & editing, Validation, Resources, Funding acquisition, Conceptualization. **Chi Zhang:** Writing – review & editing, Supervision, Resources, Project administration, Methodology, Funding acquisition, Conceptualization.

## Funding

This work was supported by the 10.13039/501100004735Hunan Provincial Natural Science Foundation of China (Grant Nos. 2024JJ9547 and 2025JJ80516).

## Declaration of competing interest

The authors declare that they have no known competing financial interests or personal relationships that could have appeared to influence the work reported in this paper.

## Data Availability

The disease surveillance data used in this study are publicly available from the National Bureau of Disease Control and Prevention of China (https://www.ndcpa.gov.cn/). The source code is available on GitHub (https://github.com/zhangch295/pred_HFRS_Lep).

## Appendix A Supplementary data

Supplementary material 1 Supplementary Tables S2–S4 and Supplementary Figure S1, presenting predictor definitions and predictor-set composition, optimized model configurations, training- and test-set performance, and seasonal-trend decomposition results for hemorrhagic fever with renal syndrome and leptospirosis in mainland China.

Supplementary material 2 Supplementary Table S1. Pairwise correlations and variance inflation factors before and after predictor selection using training-period data for forecasting monthly reported cases of hemorrhagic fever with renal syndrome and leptospirosis in mainland China, January 2010–June 2023.
