# Supplementary material for: Temporal epidemiology and multi-source forecasting of hemorrhagic fever with renal syndrome and leptospirosis in mainland China: An interpretable machine learning study
Source: Prev Med Rep. 2026 Sep 17;71:103643. doi: 10.1016/j.pmedr.2026.103643 (PMC13628220; doi:10.1016/j.pmedr.2026.103643)
Supplement: Supplementary file 1 [file mmc1.docx]

**Supplementary Materials**

**Temporal epidemiology and multi-source forecasting of hemorrhagic fever with renal syndrome and leptospirosis in mainland China: An interpretable machine learning study**

Meiqian Gong^1^, Yinzhu Zhou^1^, Shuilian Chen^1*^, Chi Zhang^1*^

**Affiliations**

^1^ Changsha Municipal Center for Disease Control and Prevention, Changsha 410004, China.

*Correspondence: Chi Zhang, [zhangch032@163.com](mailto:zhangch032@163.com); Shuilian Chen, 540432722@qq.com.

**Supplementary Contents**

Supplementary Table S1. Pairwise correlations and variance inflation factors before and after predictor selection using training-period data for forecasting monthly reported cases of hemorrhagic fever with renal syndrome and leptospirosis in mainland China, January 2010–June 2023.

Supplementary Table S2. Predictor definitions and composition of forecasting predictor sets for monthly reported cases of hemorrhagic fever with renal syndrome and leptospirosis in mainland China, January 2010–December 2025.

Supplementary Table S3. Optimized configurations of the best-performing forecasting models for monthly reported cases of hemorrhagic fever with renal syndrome and leptospirosis in mainland China, based on training data from January 2010 to June 2023.

Supplementary Table S4. Training- and test-set mean absolute error and coefficient of determination of forecasting models across predictor sets for monthly reported cases of hemorrhagic fever with renal syndrome and leptospirosis in mainland China, January 2010–December 2025.

Supplementary Figure S1. Seasonal-trend decomposition of monthly reported cases of hemorrhagic fever with renal syndrome and leptospirosis in mainland China, January 2010–December 2025.

Supplementary Table S1. Pairwise correlations and variance inflation factors before and after predictor selection using training-period data for forecasting monthly reported cases of hemorrhagic fever with renal syndrome and leptospirosis in mainland China, January 2010–June 2023. This table is provided as a separate Excel file.

Supplementary Table S2. Predictor definitions and composition of forecasting predictor sets for monthly reported cases of hemorrhagic fever with renal syndrome and leptospirosis in mainland China, January 2010–December 2025.

| Disease | Predictor set | Number of predictors | Predictors | Description |
| --- | --- | --- | --- | --- |
| Hemorrhagic fever with renal syndrome (HFRS) | M0  (historical predictors) | 8 | month sin  month cos  Incidence 1-month lag  Incidence 2-month lag  Incidence 3-month lag  Incidence 6-month lag  Incidence 12-month lag  Incidence 12-month rolling mean | sine term of calendar month  cosine term of calendar month  HFRS case, lag 1 month(s)  HFRS case, lag 2 month(s)  HFRS case, lag 3 month(s)  HFRS case, lag 6 month(s)  HFRS case, lag 12 month(s)  12-month rolling mean of prior HFRS case |
|  | M1  (historical and socioeconomic predictors) | 14 | month sin  month cos  Incidence 1-month lag  Incidence 2-month lag  Incidence 3-month lag  Incidence 6-month lag  Incidence 12-month lag  Incidence 12-month rolling mean  Agricultural gross domestic product, 1-quarter lag  Grain producer price index, 1-quarter lag  Hog producer price index, 1-quarter lag  Urban rate, 1-year lag  Population aged 0–14 years, 1-year lag  Public health institution counts, 1-year lag | sine term of calendar month  cosine term of calendar month  HFRS case, lag 1 month(s)  HFRS case, lag 2 month(s)  HFRS case, lag 3 month(s)  HFRS case, lag 6 month(s)  HFRS case, lag 12 month(s)  12-month rolling mean of prior HFRS counts  agricultural gross domestic product, lag 1 quarter  grain producer price index, lag 1 quarter  hog producer price index, lag 1 quarter  urbanization rate, lag 1 year  percentage of population aged 0–14 years lag 1 year  number of public health institutions, lag 1 year |
|  | M2  (historical and meteorological predictors) | 12 | month sin  month cos  Incidence 1-month lag  Incidence 2-month lag  Incidence 3-month lag  Incidence 6-month lag  Incidence 12-month lag  Incidence 12-month rolling mean  Temperature variance, 1-month lag  Relative humidity variance, 1-month lag  Mean relative humidity, 3-month lag  Mean precipitation, 1-month lag | sine term of calendar month  cosine term of calendar month  HFRS cases, lag 1 month(s)  HFRS cases, lag 2 month(s)  HFRS cases, lag 3 month(s)  HFRS cases, lag 6 month(s)  HFRS cases, lag 12 month(s)  12-month rolling mean of prior HFRS cases  variance of monthly mean temperature, lag 1 month  variance of monthly relative humidity, lag 1 month  monthly mean relative humidity, lag 3 months  monthly mean precipitation, lag 1 month |
|  | M3  (historical and ecological predictors) | 11 | month sin  month cos  Incidence 1-month lag  Incidence 2-month lag  Incidence 3-month lag  Incidence 6-month lag  Incidence 12-month lag  Incidence 12-month rolling mean  Nighttime light, 1-month lag  Forest coverage, 1-year lag  Disaster-affected area, 1-year lag | sine term of calendar month  cosine term of calendar month  HFRS cases, lag 1 month(s)  HFRS cases, lag 2 month(s)  HFRS cases, lag 3 month(s)  HFRS cases, lag 6 month(s)  HFRS cases, lag 12 month(s)  12-month rolling mean of prior HFRS cases  population-weighted nighttime light, lag 1 month  forest coverage percentage, lag 1 year  area affected by floods, disasters, lag 1 year |
|  | M4  (historical and behavioral predictors) | 18 | month sin  month cos  Incidence 1-month lag  Incidence 2-month lag  Incidence 3-month lag  Incidence 6-month lag  Incidence 12-month lag  Incidence 12-month rolling mean  Statutory holidays  Public holidays  School holidays  Passenger volume lag 1  Baidu search index, Hemorrhagic fever, 1-month lag  Baidu search index, Hantavirus, 1-month lag  Baidu search index, Floods, 1-month lag  Baidu search index, Epidemic hemorrhagic fever, 1-month lag  Baidu search index, Leptospirosis, 1-month lag  Baidu search index, Rodent, 1-month lag | sine term of calendar month  cosine term of calendar month  HFRS cases, lag 1 month(s)  HFRS cases, lag 2 month(s)  HFRS cases, lag 3 month(s)  HFRS cases, lag 6 month(s)  HFRS cases, lag 12 month(s)  12-month rolling mean of prior HFRS cases  number of statutory holiday days in the month  number of public holiday days in the month  number of school holiday days in the month  total passenger volume, lag 1 month  Baidu Index for ‘hemorrhagic fever’, lag 1 month  Baidu Index for ‘Hantavirus’, lag 1 month  Baidu Index for ‘floods’, lag 1 month  Baidu Index for ‘epidemic hemorrhagic fever’, lag 1 month  Baidu Index for ‘leptospirosis’, lag 1 month  Baidu Index for ‘rodent infestation’, lag 1 month |
|  | M5  (all predictor domains) | 26 | Incidence 1-month lag  Incidence 2-month lag  Incidence 3-month lag  Incidence 6-month lag  Incidence 12-month lag  Incidence 12-month rolling mean  Agricultural gross domestic product, 1-quarter lag  Grain producer price index, 1-quarter lag  Hog producer price index, 1-quarter lag  Population aged 0–14 years, 1-year lag  Public health institution counts, 1-year lag  Temperature variance, 1-month lag  Relative humidity variance, 1-month lag  Mean relative humidity, 3-month lag  Mean precipitation, 1-month lag  Nighttime light, 1-month lag  Forest coverage, 1-year lag  Disaster-affected area, 1-year lag  Statutory holidays  Public holidays  School holidays  Baidu search index, Hemorrhagic fever, 1-month lag  Baidu search index, Hantavirus, 1-month lag  Baidu search index, Floods, 1-month lag  Baidu search index, Epidemic hemorrhagic fever, 1-month lag  Baidu search index, Rodent, 1-month lag | HFRS cases, lag 1 month(s)  HFRS cases, lag 2 month(s)  HFRS cases, lag 3 month(s)  HFRS cases, lag 6 month(s)  HFRS cases, lag 12 month(s)  12-month rolling mean of prior HFRS cases  agricultural gross domestic product, lag 1 quarter  grain producer price index, lag 1 quarter  hog producer price index, lag 1 quarter  percentage of population aged 0–14 years, lag 1 year  number of public health institutions, lag 1 year  variance of monthly mean temperature, lag 1 month  variance of monthly relative humidity, lag 1 month  monthly mean relative humidity, lag 3 months  monthly mean precipitation, lag 1 month  population-weighted nighttime light, lag 1 month  forest coverage percentage, lag 1 year  area affected by floods, disasters, lag 1 year  number of statutory holiday days in the month  number of public holiday days in the month  number of school holiday days in the month  Baidu Index for ‘hemorrhagic fever’, lag 1 month  Baidu Index for ‘Hantavirus’, lag 1 month  Baidu Index for ‘floods’, lag 1 month  Baidu Index for ‘epidemic hemorrhagic fever’, lag 1 month  Baidu Index for ‘rodent infestation’, lag 1 month |
| Leptospirosis | M0  (historical predictors) | 8 | month sin  month cos  Incidence 1-month lag  Incidence 2-month lag  Incidence 3-month lag  Incidence 6-month lag  Incidence 12-month lag  Incidence 12-month rolling mean | sine term of calendar month  cosine term of calendar month  Leptospirosis cases, lag 1 month(s)  Leptospirosis cases, lag 2 month(s)  Leptospirosis cases, lag 3 month(s)  Leptospirosis cases, lag 6 month(s)  Leptospirosis cases, lag 12 month(s)  12-month rolling mean of prior Leptospirosis cases |
|  | M1  (historical and socioeconomic predictors) | 14 | month sin  month cos  Incidence 1-month lag  Incidence 2-month lag  Incidence 3-month lag  Incidence 6-month lag  Incidence 12-month lag  Incidence 12-month rolling mean  Agricultural gross domestic product, 1-quarter lag  Grain producer price index, 1-quarter lag  Hog producer price index, 1-quarter lag  Urban rate, 1-year lag  Population aged 0–14 years, 1-year lag  Public health institution counts, 1-year lag | sine term of calendar month  cosine term of calendar month  Leptospirosis cases, lag 1 month(s)  Leptospirosis cases, lag 2 month(s)  Leptospirosis cases, lag 3 month(s)  Leptospirosis cases, lag 6 month(s)  Leptospirosis cases, lag 12 month(s)  12-month rolling mean of prior Leptospirosis cases  agricultural gross domestic product, lag 1 quarter  grain producer price index, lag 1 quarter  hog producer price index, lag 1 quarter  urbanization rate, lag 1 year  percentage of population aged 0–14 years lag 1 year  number of public health institutions, lag 1 year |
|  | M2  (historical and meteorological predictors) | 13 | month cos  Incidence 1-month lag  Incidence 2-month lag  Incidence 3-month lag  Incidence 6-month lag  Incidence 12-month lag  Incidence 12-month rolling mean  Temperature variance, 1-month lag  Mean relative humidity, 1-month lag  Relative humidity variance, 1-month lag  Mean relative humidity, 3-month lag  Precipitation variance, 1-month lag  Mean precipitation, 3-month lag | cosine term of calendar month  Leptospirosis cases, lag 1 month(s)  Leptospirosis cases, lag 2 month(s)  Leptospirosis cases, lag 3 month(s)  Leptospirosis cases, lag 6 month(s)  Leptospirosis cases, lag 12 month(s)  12-month rolling mean of prior Leptospirosis cases  variance of monthly mean temperature, lag 1 month  monthly mean relative humidity, lag 1 month  variance of monthly relative humidity, lag 1 month  monthly mean relative humidity, lag 3 months  variance of monthly precipitation, lag 1 month  monthly mean precipitation, lag 3 months |
|  | M3  (historical and ecological predictors) | 11 | month sin  month cos  Incidence 1-month lag  Incidence 2-month lag  Incidence 3-month lag  Incidence 6-month lag  Incidence 12-month lag  Incidence 12-month rolling mean  Nighttime light, 1-month lag  Forest coverage, 1-year lag  Disaster-affected area, 1-year lag | sine term of calendar month  cosine term of calendar month  Leptospirosis cases, lag 1 month(s)  Leptospirosis cases, lag 2 month(s)  Leptospirosis cases, lag 3 month(s)  Leptospirosis cases, lag 6 month(s)  Leptospirosis cases, lag 12 month(s)  12-month rolling mean of prior Leptospirosis cases  population-weighted nighttime light, lag 1 month  forest coverage percentage, lag 1 year  area affected by floods, disasters, lag 1 year |
|  | M4  (historical and behavioral predictors) | 18 | month sin  month cos  Incidence 1-month lag  Incidence 2-month lag  Incidence 3-month lag  Incidence 6-month lag  Incidence 12-month lag  Incidence 12-month rolling mean  Statutory holidays  Public holidays  School holidays  Passenger volume lag 1  Baidu search index, Hemorrhagic fever, 1-month lag  Baidu search index, Hantavirus, 1-month lag  Baidu search index, Floods, 1-month lag  Baidu search index, Epidemic hemorrhagic fever, 1-month lag  Baidu search index, Leptospirosis, 1-month lag  Baidu search index, Rodent, 1-month lag | sine term of calendar month  cosine term of calendar month  Leptospirosis cases, lag 1 month(s)  Leptospirosis cases, lag 2 month(s)  Leptospirosis cases, lag 3 month(s)  Leptospirosis cases, lag 6 month(s)  Leptospirosis cases, lag 12 month(s)  12-month rolling mean of prior Leptospirosis cases  number of statutory holiday days in the month  number of public holiday days in the month  number of school holiday days in the month  total passenger volume, lag 1 month  Baidu Index for ‘hemorrhagic fever’, lag 1 month  Baidu Index for ‘Hantavirus’, lag 1 month  Baidu Index for ‘floods’, lag 1 month  Baidu Index for ‘epidemic hemorrhagic fever’, lag 1 month  Baidu Index for ‘leptospirosis’, lag 1 month  Baidu Index for ‘rodent infestation’, lag 1 month |
|  | M5  (all predictor domains) | 28 | Incidence 1-month lag  Incidence 2-month lag  Incidence 3-month lag  Incidence 6-month lag  Incidence 12-month lag  Incidence 12-month rolling mean  Agricultural gross domestic product, 1-quarter lag  Grain producer price index, 1-quarter lag  Hog producer price index, 1-quarter lag  Population aged 0–14 years, 1-year lag  Public health institution counts, 1-year lag  Temperature variance, 1-month lag  Mean relative humidity, 1-month lag  Relative humidity variance, 1-month lag  Mean relative humidity, 3-month lag  Precipitation variance, 1-month lag  Mean precipitation, 3-month lag  Nighttime light, 1-month lag  Forest coverage, 1-year lag  Disaster-affected area, 1-year lag  Statutory holidays  Public holidays  School holidays  Baidu search index, Hemorrhagic fever, 1-month lag  Baidu search index, Hantavirus, 1-month lag  Baidu search index, Floods, 1-month lag  Baidu search index, Leptospirosis, 1-month lag  Baidu search index, Rodent, 1-month lag | Leptospirosis cases, lag 1 month(s)  Leptospirosis cases, lag 2 month(s)  Leptospirosis cases, lag 3 month(s)  Leptospirosis cases, lag 6 month(s)  Leptospirosis cases, lag 12 month(s)  12-month rolling mean of prior Leptospirosis cases  agricultural gross domestic product, lag 1 quarter  grain producer price index, lag 1 quarter  hog producer price index, lag 1 quarter  percentage of population aged 0–14 years, lag 1 year  number of public health institutions, lag 1 year  variance of monthly mean temperature, lag 1 month  monthly mean relative humidity, lag 1 month  variance of monthly relative humidity, lag 1 month  monthly mean relative humidity, lag 3 months  variance of monthly precipitation, lag 1 month  monthly mean precipitation, lag 3 months  population-weighted nighttime light, lag 1 month  forest coverage percentage, lag 1 year  area affected by floods, disasters, lag 1 year  number of statutory holiday days in the month  number of public holiday days in the month  number of school holiday days in the month  Baidu Index for ‘hemorrhagic fever’, lag 1 month  Baidu Index for ‘Hantavirus’, lag 1 month  Baidu Index for ‘floods’, lag 1 month  Baidu Index for ‘leptospirosis’, lag 1 month  Baidu Index for ‘rodent infestation’, lag 1 month |

Note: The number of predictors indicates the total included in each disease-specific predictor set. M0 included historical predictors only; M1, M2, M3, and M4 extended M0 with socioeconomic, meteorological, ecological, and behavioral predictors, respectively; and M5 combined selected predictors from all five domains. Annual and quarterly indicators were converted to monthly series by assigning the reported value to all months within the corresponding reporting period. To ensure data availability at each one-month-ahead forecast origin, previous-year and previous-quarter values were represented using 13-month and 4-month shifts, respectively. HFRS, hemorrhagic fever with renal syndrome.

Supplementary Table S3. Optimized configurations of the best-performing forecasting models for monthly reported cases of hemorrhagic fever with renal syndrome and leptospirosis in mainland China, based on training data from January 2010 to June 2023.

| Disease | Model | Optimized hyperparameters |
| --- | --- | --- |
| Hemorrhagic fever with renal syndrome | Seasonal autoregressive integrated moving average (SARIMA)  Historical predictors (M0) | SARIMA (1,0,1)(0,1,1)_12_; trend:n; enforce_stationarity:False; enforce_invertibility:False; Method: lbfgs; |
|  | Random forest  All predictor domains (M5) | n_estimators: 4; max_depth: 5; min_samples_leaf: 1; min_samples_split: 4; max_features: 0.77 |
|  | Extreme gradient boosting (XGBoost)  All predictor domains (M5) | n_estimators : 21; learning_rate:0.3; subsample:0.66; colsample_bytree:1; reg_alpha:0; reg_lambda:1; gamma: 0; max_depth:2; min_child_weight:1 |
|  | Long short-term memory (LSTM)  Historical predictors (M0) | hidden_size: 16; num_layers: 1; input_dim: 8; dropout: 0.2; weight_decay: 0.0001; batch_size: 16; learning_rate: 0.001; optimizer: Adam; loss: MSE Loss; lookback: 12 |
|  | Temporal convolutional network (TCN)  Historical and meteorological predictors (M2) | num_channels: [8, 8]; kernel_size: 3; input_dim: 12; dropout: 0.2; weight_decay: 0.0001; batch_size: 8; learning_rate: 0.001; optimizer: Adam; loss: MSELoss; lookback: 12; |
| Leptospirosis | Seasonal autoregressive integrated moving average (SARIMA)  Historical predictors (M0) | SARIMA (0,1,1)(0,1,1)_12_; trend:n; enforce_stationarity:False; enforce_invertibility:False; Method: lbfgs |
|  | Random forest  All predictor domains (M5) | n_estimators:3; max_depth: 10; min_samples_leaf: 1; min_samples_split: 2; max_features: 0.46 |
|  | Extreme gradient boosting (XGBoost)  All predictor domains (M5) | n_estimators : 59; learning_rate:0.28; subsample:0.42; colsample_bytree:1; gamma: 0.15; reg_alpha:0; reg_lambda:1; max_depth:2; min_child_weight:1 |
|  | Long short-term memory (LSTM)  Historical and meteorological predictors (M2) | hidden_size: 16; num_layers: 1; input_dim: 13; dropout: 0.2; weight_decay: 0.0001; batch_size: 16; learning_rate: 0.001; optimizer: Adam; loss: MSE Loss; lookback: 12 |
|  | Temporal convolutional network (TCN)  Historical and meteorological predictors (M2) | num_channels: [16, 16, 16]; kernel_size: 2; input_dim: 13; dropout: 0.2; weight_decay: 0.0001; batch_size: 8; learning_rate: 0.001; optimizer: Adam; loss: MSELoss; lookback: 12; |

Note: M0 included historical predictors only; M2 included historical and meteorological predictors; and M5 included all five predictor domains. Seasonal autoregressive integrated moving average was evaluated only under M0. SARIMA, seasonal autoregressive integrated moving average; XGBoost, extreme gradient boosting; LSTM, long short-term memory; TCN, temporal convolutional network.

Supplementary Table S4. Training- and test-set mean absolute error and coefficient of determination of forecasting models across predictor sets for monthly reported cases of hemorrhagic fever with renal syndrome and leptospirosis in mainland China, January 2010–December 2025.

| Disease | Model | Dataset | M0  (historical predictors) | M1  (historical and socioeconomic predictors) | M2  (historical and meteorological predictors) | M3  (historical and ecological predictors) | M4  (historical and behavioral predictors) | M5  (all predictor domains) |
| --- | --- | --- | --- | --- | --- | --- | --- | --- |
| Hemorrhagic fever with renal syndrome | Seasonal autoregressive integrated moving average | Training | 125.71 (0.87) | — | — | — | — | — |
|  |  | Test | 124.42 (0.53) | — | — | — | — | — |
|  | Random forest | Training | 102.98 (0.88) | 105.92 (0.88) | 83.00 (0.91) | 107.78 (0.87) | 90.62 (0.90) | 91.66 (0.92) |
|  |  | Test | 112.19 (0.53) | 122.26 (0.58) | 111.90 (0.64) | 118.09 (0.53) | 108.17 (0.58) | 109.66 (0.69) |
|  | Extreme gradient boosting | Training | 122.97 (0.88) | 100.13 (0.92) | 81.87 (0.94) | 101.12 (0.91) | 110.62 (0.90) | 76.43 (0.95) |
|  |  | Test | 110.19 (0.55) | 117.13 (0.61) | 92.19 (0.76) | 116.45 (0.57) | 120.35 (0.60) | 93.94 (0.79) |
|  | Long short-term memory | Training | 89.12 (0.92) | 97.36 (0.89) | 76.95 (0.92) | 84.33 (0.91) | 113.67 (0.86) | 113.35 (0.86) |
|  |  | Test | 152.04 (0.56) | 404.20 (-1.62) | 185.56 (0.17) | 200.86 (0.21) | 325.14 (-0.69) | 410.40 (-1.81) |
|  | Temporal convolutional network | Training | 61.89 (0.95) | 71.04 (0.91) | 55.49 (0.96) | 56.60 (0.96) | 74.77 (0.91) | 102.21 (0.89) |
|  |  | Test | 166.84 (0.46) | 351.22 (-1.06) | 103.36 (0.62) | 148.59 (0.56) | 134.75 (0.60) | 259.25 (-0.18) |
| Leptospirosis | Seasonal autoregressive integrated moving average | Training | 14.60 (0.29) | — | — | — | — | — |
|  |  | Test | 19.11 (0.68) | — | — | — | — | — |
|  | Random forest | Training | 7.15 (0.74) | 7.16 (0.75) | 5.84 (0.80) | 7.34 (0.75) | 6.11 (0.80) | 5.80 (0.81) |
|  |  | Test | 19.73 (0.57) | 17.29 (0.63) | 15.58 (0.72) | 19.14 (0.59) | 16.47 (0.68) | 14.87 (0.80) |
|  | Extreme gradient boosting | Training | 6.21 (0.79) | 6.30 (0.84) | 4.56 (0.88) | 6.44 (0.79) | 5.48 (0.83) | 4.52 (0.92) |
|  |  | Test | 17.71 (0.66) | 15.72 (0.72) | 14.30 (0.82) | 14.81 (0.71) | 13.59 (0.80) | 10.88 (0.91) |
|  | Long short-term memory | Training | 9.64 (0.56) | 11.28 (0.48) | 8.73 (0.62) | 10.34 (0.51) | 8.73 (0.62) | 7.69 (0.69) |
|  |  | Test | 22.45 (0.43) | 29.28 (0.06) | 19.70 (0.59) | 28.42 (0.12) | 27.05 (0.14) | 33.31 (-0.20) |
|  | Temporal convolutional network | Training | 9.28 (0.58) | 10.30 (0.51) | 7.20 (0.68) | 11.30 (0.47) | 8.11 (0.66) | 9.82 (0.53) |
|  |  | Test | 22.13 (0.50) | 24.89 (0.29) | 19.94 (0.59) | 26.79 (0.17) | 24.81 (0.27) | 21.81 (0.42) |

Note: Values are presented as mean absolute error (MAE), with the coefficient of determination (R²) in parentheses [MAE (R²)]. Lower MAE and higher R² indicate better predictive performance; negative R² values indicate performance worse than a model that always predicts the observed mean. Training-set metrics excluded the initial 12-month burn-in period, whereas test-set metrics were calculated from 30 rolling one-month-ahead forecasts from July 2023 to December 2025. M0 included historical predictors only; M1, M2, M3, and M4 extended M0 with socioeconomic, meteorological, ecological, and behavioral predictors, respectively; and M5 combined selected predictors from all five domains. Seasonal autoregressive integrated moving average was evaluated only under M0; dashes indicate not applicable.

**Supplementary Figure S1. Seasonal-trend decomposition of monthly reported cases of hemorrhagic fever with renal syndrome and leptospirosis in mainland China, January 2010–December 2025**. (A) Seasonal-trend decomposition using locally estimated scatterplot smoothing (STL) for hemorrhagic fever with renal syndrome (HFRS). (B) Corresponding decomposition for leptospirosis. For each disease, the observed series was decomposed into trend, seasonal, and remainder components. The observed and trend components are shown as monthly case counts, whereas the seasonal and remainder components are shown on a logarithmic scale. Horizontal dashed lines indicate zero. HFRS showed a more pronounced long-term trend, whereas leptospirosis showed a stronger recurring seasonal component.
